# Changes in the human footprint in and around Indonesia’s terrestrial national parks between 2012 and 2017

**DOI:** 10.1038/s41598-021-83586-2

**Published:** 2021-02-24

**Authors:** Asri A. Dwiyahreni, Habiburrachman A. H. Fuad, Sunaryo Muhtar, T. E. Budhi Soesilo, Chris Margules, Jatna Supriatna

**Affiliations:** 1 School of Environmental Science, Universitas Indonesia, Jakarta, 10430 Indonesia; 2 Department Biology, Faculty of Mathematics and Natural Sciences, Universitas Indonesia, Depok, 16424 Indonesia; 3 Research Center for Climate Change, Faculty of Mathematics and Natural Sciences, Universitas Indonesia, Depok, 16424 Indonesia; 4 Faculty of Mathematics and Natural Sciences, Institute for Sustainable Earth and Resources, Universitas Indonesia, Depok, 16424 Indonesia; 5 Centre for Tropical Environmental Sustainability Science, College of Science and Engineering, James Cook University, Cairns, Australia

**Keywords:** Ecology, Environmental sciences

## Abstract

The human footprint (HF) was developed to measure of the impact of human activities on the environment. The human footprint has been found to be closely related to the vulnerability of protected areas around the world. In Indonesia, as nature conservation is still seen as hindering economic development, it is especially important to assess the human footprint in order to comprehend the overall pressures resulting from the various human activities on Indonesia’s national parks. This study measured the change in the human footprint in and around 43 terrestrial national parks over 5 years, between 2012 and 2017. As many as 37 out of 43 NPs experienced an increase in the HF, ranging from 0.4 to 77.3%. Tanjung Puting in Kalimantan experienced the greatest increase (77.3%), while Ujung Kulon in Jawa Bali bioregion had the greatest decrease (10.5%). An increase in human population density and improved access to parks from roads, rivers and coastlines are the main drivers of increasing impacts on national parks.

## Introduction

Indonesia is the largest island nation in the world with 17,504 islands covering a land area of 1.91 million km^2^^1^. More than 60% has forest status, not all of which is actually forested, controlled by the state^2^. Of this, more than 22 million ha or 21.26% is managed as protected areas (PAs)^3^. This exceeds the area of PAs in most countries in Asia, Africa and Latin America^4^. Indonesia has already exceeded the Aichi Biodiversity Targets aim of protecting 13% to 17% of the land surface by 2020^5,6^. However, human activities may have an impact on PAs, decreasing their effectiveness. In Indonesia, current data show that approximately 2.2 million ha of degraded lands occur in terrestrial PAs. This is approximately 10% of their total area^7^. Amid the current high pressures for economic and human development, forest ecosystems, wildlife populations and the various ecosystem services that they provide will likely only remain in protected areas (PAs). Protected areas have become the lead efforts of nature conservation^8^. A study of global PAs showed that PAs were able to protect habitats from deforestation^9^. Forest cover in Sumatra, Indonesia between 2000 and 2012 was more intact in and around national parks^10^. This was also the case for biodiversity; species richness was found to be 10.6% and abundance 14.5% higher inside than outside of PAs globally^11^. However, around 10% of global PAs are ‘paper parks’, protected areas with legal status that do not have management activities on the ground. More than 60% only have basic support with a significant lack of management activities^12^. Protected areas that lack conservation management capability tend to suffer from habitat and biodiversity loss^13,14^. PAs in Indonesia have not yet been proven to protect forests and biodiversity. The rate of deforestation in PAs in Sumatra is no different to that in production forest areas. Protected areas are no more effective in maintaining forest cover than areas managed for timber production^15^. The population of Sumatran elephants is estimated to have dropped by 35% since the 1990s^16^. Likewise, populations of Sulawesi's endemic species^17^ such as the anoa^18^, continue to decline. Sumatran primates^10,19^ and tigers^20^ also have not been able to maintain their populations within PAs.

This study examined pressures caused by human activities in and around terrestrial national parks in Indonesia between 2012 and 2017. In 2015, there were changes in the management of Indonesia’s PAs. Previous to 2015, PAs were managed under the Directorate General of Forest Protection and Nature Conservation (Direktorat Jenderal Perlindungan Hutan dan Konservasi Alam – PHKA), Ministry of Forestry. In 2015 this was changed to the Directorate General of Natural Resource and Ecosystems Conservation (Direktorat Jenderal Konservasi Sumber Daya Alam dan Ekosistem – KSDAE), Ministry of Environment and Forestry. The major difference is that law enforcement power used to be within the directorate general PHKA itself, whereas following this change KSDAE must work with a different directorate general within the Ministry of environment and forestry for law enforcement. Due to these changes, the number of forest guards in each park was reduced by 32% in 2017 compared to 2012^21^. This study also includes an assessment of the impact of these changes on the degree of human pressures on national parks. Hence, for comparability reasons, this study only examined the 43 terrestrial NPs established up to and including 2012. All 43 of these NPs have their own management units. The four NPs that are not included in this study are new and do not yet have separate management units.

Pressures from human activities were calculated using Human Footprint (HF) analysis. The Human Influence Index (HII) was developed to measure anthropogenic impacts on the environment. The resultant map is called a Human Footprint map^22^. It can be used, among other things, for conservation planning, developing strategies for natural resource management and studies on human—environment interactions^23^. Previous studies have shown that the HF from economic and associated human development activities increases the vulnerability of PAs around the world^24,25^. In this study, we map the HII score in and around Indonesian national parks in 2012 and 2017 as the Human Footprint. The score is standardized per hectare for comparisons among NPs and bioregions. Percent changes of HII score per ha between 2012 and 2017 in each NP were also calculated to assess whether they were changing or not, and if they were, to discuss options for limiting their effects. Other HF studies in Mexico^26^, Ecuador^27^ and Tibet^28^ have shown that the HF has caused natural habitat degradation even inside protected areas, and even where unfavorable physical conditions such as high elevation and steep terrain hinders human activities^29,30^.

Indonesia currently has 54 national parks (NPs), which are a sub-set of PAs. There are 47 terrestrial NPs and 7 marine NPs. Among those, 27 have international status as World Heritage, Biosphere Reserve, ASEAN Heritage, or Ramsar Convention Sites^31^. In Indonesia, NPs have the most well-resourced management regimes compared to other kinds of PAs, such as Nature Reserve (NR) or Wildlife Reserve (WR). Each NP has its own management unit, while several NRs or WRs may be managed by a single management unit. Indonesia’s NPs cover approximately 16 million ha, or 60% of the total area covered by PAs in Indonesia^7^. They also contain 6,861,497.92 ha of primary forests, which increased by 0.07% between 2012 and 2017^21^.

## Results

### Drivers of the human footprint in and around national parks

**Figure 1:**
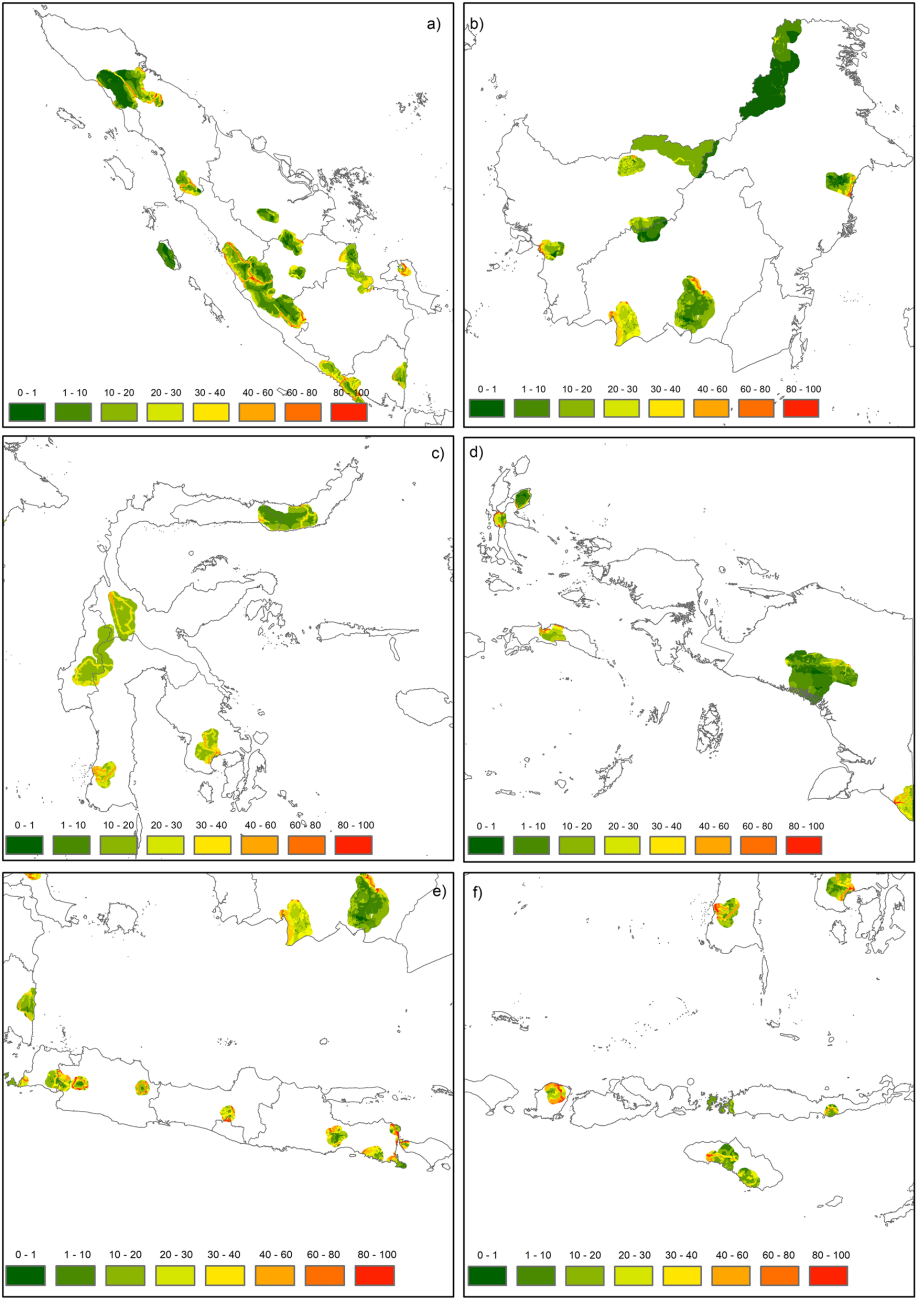
Human Footprint in 2017 within and around 43 terrestrial national parks (NPs) in 7 bioregions in Indonesia: (**a**) Sumatra, (**b**) Kalimantan, (**c**) Sulawesi, (**d**) Maluku and Papua, (**e**) Jawa and Bali, and (**f**) Nusa Tenggara. Activity data maps of Indonesian NPs (land cover maps, roads, river and settlements) used in the Human Footprint analysis in this study were obtained from the Indonesian Ministry of Environment and Forestry^32^, and Binamarga^33^. National Parks boundaries were obtained from The World Database on Protected Areas (https://www.protectedplanet.net/en). The Human Footprint maps were created using Esri ArcMap 10.8 (https://www.arcgis.com).

**Figure 2:**
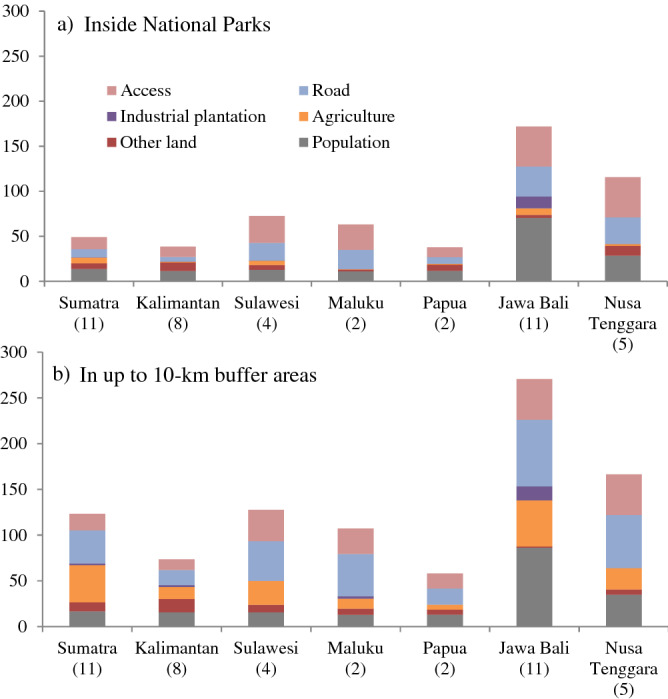
Each Human Footprint pressure per hectare NPs areas in 2017 in 7 bioregions in Indonesia. The numbers below each bioregion are the number of NPs in that bioregion.

Human Footprint (HF) in Indonesia’s national parks (NPs) and their buffer areas were calculated for the years 2012 and 2017 to see if there are any significant difference across those 5 years, and if so, whether they could be related to the organizational changes in the Ministry of Environment and Forestry in 2015. Difference between 2012 and 2017 were detected but were too subtle to be shown on a map at the scale used here, so only the 2017 map is presented here. The Human Footprint 2017 map shows that Jawa Bali has the highest HF pressures both inside the parks and in the 10-km buffer areas, is followed by Nusa Tenggara. National parks in Sulawesi, Maluku, Sumatra, Kalimantan and Papua have lower HF pressures than those in Jawa Bali and Nusa Tenggara (Fig. 1). Human Footprint pressures inside the 43 terrestrial NPs and their buffer areas in 2017 varied greatly. Some bioregions had high HF while the others were relatively low, and all bioregions experienced higher HFs in the buffer areas than inside the parks (Fig. 2). This HF analysis for areas inside and around Indonesia’s terrestrial NPs is valid. The validation analysis showed that the Root Mean Square Error (RMS Error) for HF scoring was 0.14 and 0.13 for 2012 and 2017, respectively. This means there were errors around 13% and 14% in assigning HF measures and scoring pressures compared to the visual interpretation of the high resolution map. The Kappa Statistic analyses for 2012 and 2017 data were both 0.65 (*P* < 0.05). The Human Footprint assigned scores were a match to the visual scores if they were more than 0.5 or within 20% of one another on the 0–1 scale.

In this study, HF is a summation of pressures from human population, roads, access (from roads, railways, big rivers and coastlines), settlements, and land transformation. Land transformation comprises agriculture, industrial plantation (IP), built up environment (BUE) and other land uses (OLU) such as bare lands and shrubs (Table 2 in the “Methods” section). In total, inside the parks, access delivers the highest HF, followed by human population, roads and land transformation. Outside the parks, roads provide the highest HF, followed by land transformation, access, and human population. Human Footprint also calculated the score of settlements and built up environments both inside the parks and in their buffer areas, however their scores are very low compared to the other pressures, ranging from 0 to 0.1 in each bioregion, hence are not presented (Fig. 2).

In Sumatra population is the highest pressure inside NPs, followed by access, roads, agriculture, OLU, and industrial plantation. In the 10-km buffer areas, agriculture is the highest HF driver, followed by roads, access, population, OLU, and IP. In Kalimantan, population is not the highest pressure within NPs, access is. Other land use comes next, followed by roads, agriculture and IP. Outside NPs, roads provide the highest HF, followed by population, OLU, access, agriculture, and IP. In Sulawesi, NPs are pressured by access and roads, then by population, OLU, agriculture and IP. In their buffer areas, roads and access are the two highest pressures. Agriculture follows then population, OLU and IP (Fig. 2).

Maluku also experiences access and roads as the highest provider of HF, followed by population, OLU and agriculture. In the buffer areas, roads provide the highest HF, followed by access, population, agriculture, OLU, and IP (Fig. 2).

**Figure 3:**
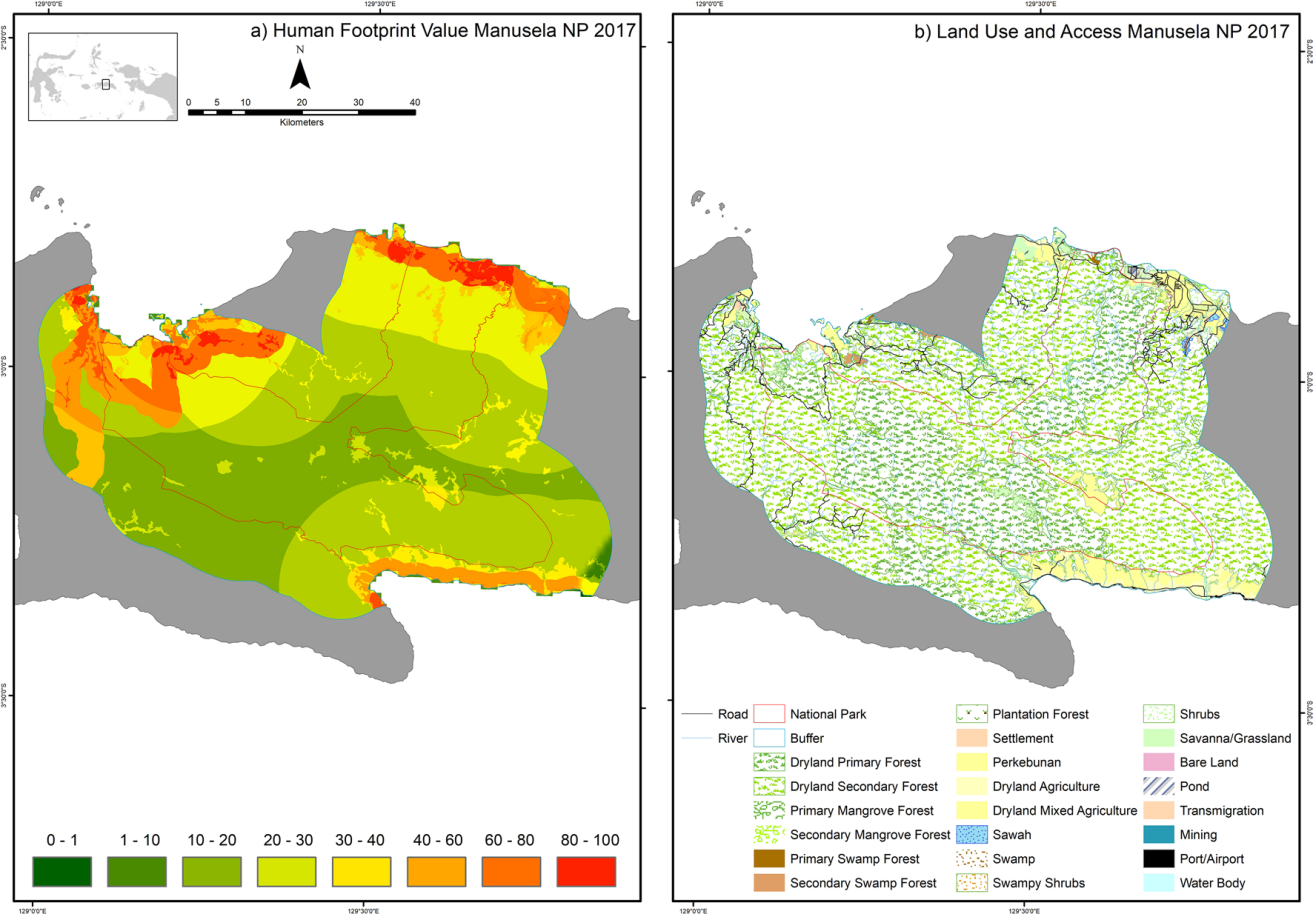
Human Footprint and land use in Manusela National Park, Maluku in 2017. Activity data maps of Manusela NP (land cover maps, roads, river and settlements) used in the Human Footprint analysis in this study were obtained from the Indonesian Ministry of Environment and Forestry^32^ and Binamarga^33^ and NP boundary was obtained from The World Database on Protected Areas (https://www.protectedplanet.net/en). The Maps were created using Esri ArcMap 10.8 (https://www.arcgis.com).

The highest HF per ha of any NP in Indonesia occurred in Manusela NP. Approximately 75% of the area of Manusela NP is in Central Maluku District, Maluku Province. Central Maluku District is a focus for agricultural development in Maluku because it is suitable for the expansion of rice paddy fields to support Maluku’s food security program^34^. In addition, Maluku is currently undergoing a trans Maluku roads development program including on Seram Island where Manusela NP is located. These two factors together have had a high impact on Manusela NP and its buffer zone (Fig. 3).

Papua bioregion has the lowest HF inside and outside its NPs, while Nusa Tenggara has the second highest scores for HF after the Jawa Bali bioregion. NPs in Papua experience the highest HF from population, then access, roads, OLU, and agriculture. Outside the parks, it experiences the highest HF from roads then access, population, agriculture, and OLU. In Nusa Tenggara, access and roads cause the highest HF in NPs, followed by population, OLU, agriculture, and IP. In buffer areas, highest HF also comes from roads and access, followed by population, agriculture, OLU, BUE, and IP (Fig. 2).

### Changes in the human footprint between 2012 and 2017

**Figure 4:**
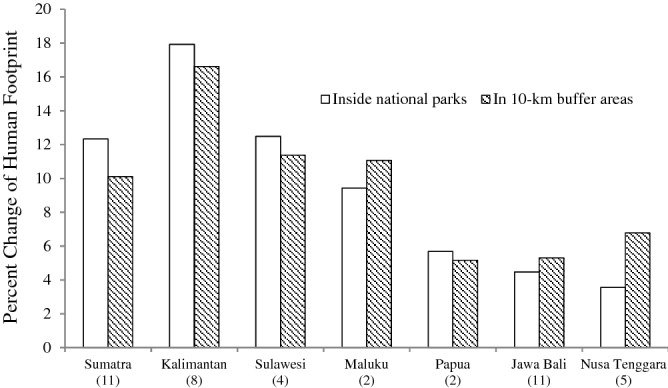
Average percent changes of the Human Footprint in the 7 bioregion of Indonesia between 2012 and 2017.

In order to plan effective protected areas management programs, it is important to see how pressures change over time, especially in a relatively short periods. This study shows that the HF has changed in and around NPs in Indonesia, even in a period of only 5 years. Between 2012 and 2017, all bioregions experienced average increases in their HFs, both inside parks as well as in the 10-km buffer areas outside the parks. The results of regression analysis show a causal significant relationship (R^2^ = 0.53, df = 33, *P* < 0.01) between the HF in NPs buffer areas and HF inside NPs. When the HF outside increases, then the HF inside also increases. Inside the parks, the highest HF increases occurred in the Kalimantan bioregion, where the average changes across eight NPs was 17.93%. The lowest increases occurred in the Nusa Tenggara bioregion, with an average change of 3.56% in five NPs. In the 10-km buffer areas, the highest increase also occurred in Kalimantan with an average of 16.61%. The lowest occurred in the Papua bioregion with an average change of 5.16% from two NPs. On average, more bioregions in Indonesia experienced higher increases of HFs inside NP compared to HFs in the 10-km buffer areas (Sumatra, Kalimantan, Sulawesi, and Papua). But HFs in the buffer areas in Nusa Tenggara, Jawa Bali and Maluku increased more than in the NPs (Fig. 4).

Individually, some NPs had relatively low HF changes, while others had relatively high changes. Ujung Kulon NP in the Jawa Bali bioregion, experienced a decrease of 10.5% over the 5 years between 2012 and 2017. Five other NPs, Alas Purwo, Baluran and Bali Barat also in Jawa Bali, Danau Sentarum in Kalimantan, and Komodo in Nusa Tenggara also experienced a decrease inside the parks. Those four NPs in the Jawa Bali bioregion also had a decrease within their 10-km buffer areas, while Danau Sentarum and Komodo experienced increases in their buffer areas (Table 1).

**Table 1:** Changes in Human Footprint (Human Influence Index scores), inside and outside 43 terrestrial national parks in Indonesia between 2012 to 2017.

Bioregion and national parks (NPs)	NP terrestrial area (ha)	% Change of human influence index (HII) per ha	
		Inside NP	Outside NP (10 km buffer areas)
**Sumatra (11 NPs)**			
Batang Gadis	96,207.84	11.3	11.4
Berbak	193,886.46	13.9	13.8
Bukit Barisan Selatan	384,371.19	11.4	11.9
Bukit Dua Belas	68,108.85	3.3	7.6
Bukit Tiga Puluh	179,270.64	12.1	9
Gunung Leuser	1,191,509.73	10.1	10.7
Kerinci Seblat	1,715,978.34	12	11.7
Sembilang	257,749.38	6.9	8.6
Siberut	237,472.38	11.2	11.7
Tesso Nilo	103,718.97	19.1	7.5
Way Kambas	151,808.31	24.4	7.1
**Kalimantan (8 NPs)**			
Betung Kerihun	870,712.83	13.2	11.9
Bukit Raya Bukit Baka	258,958.80	1.5	4.1
Danau Sentarum	138,393.00	− 2.4	1
Gunung Palung	114,968.16	11.4	11.9
Kayan Mentarang	1,359,825.84	14.3	14.5
Kutai	208,830.24	15.1	14
Sebangau	643,125.78	12.9	11.9
Tanjung Puting	426,586.68	77.3	63.7
**Sulawesi (4 NPs)**			
Bantimurung Bulusaraung	44,395.47	11.4	11.8
Bogani Nani Wartabone	285,306.48	11.2	11.5
Lore Lindu	223,858.89	11.2	9.8
Rawa Aopa Watumohai	107,562.06	16.1	12.5
**Maluku (2 NPs)**			
Aketajawe Lolobata	163,045.62	8.3	10.9
Manusela	174,598.65	10.6	11.2
**Papua (2 NPs)**			
Lorentz	2,239,199.73	11	10.2
Wasur	465,429.24	0.4	0.1
**Jawa Bali (11 NPs)**			
Alas Purwo	46,382.58	− 6	− 2
Bali Barat	14,758.56	− 0.6	− 2.3
Baluran	28,801.98	− 0.5	− 3.4
Bromo Tengger Semeru	54,331.83	11.4	11
Gunung Ciremai	16,809.84	8	10.9
Gunung Gede Pangrango	28,160.64	11	9.9
Gunung Halimun Salak	86,963.94	9.9	10.4
Gunung Merapi	7197.66	7.7	5.6
Gunung Merbabu	6357.69	7.2	8
Meru Betiri	56,108.61	11.6	11.7
Ujung Kulon	76,794.12	− 10.5	− 1.6
**Nusa Tenggara (5 NPs)**			
Gunung Rinjani	38,743.38	10.8	12.2
Kelimutu	5661.09	1.4	0.4
Komodo	67,014.00	− 2.3	3.5
Laiwangi Wanggameti	40,211.46	3.1	16.3
Manupeu Tanah Daru	77,626.08	4.7	1.5

All other NPs experienced an increase in the HF inside the park and in the buffer areas (Table 1). Among all NPs, Tanjung Puting in Kalimantan experienced the greatest increase inside the park (77.3%). In each bioregion, the highest increases inside parks were experienced by Way Kambas in Sumatra (24.4%), Rawa Aopa Watumohai in Sulawesi (16%), Meru Betiri in Jawa Bali (11.6%), Lorentz in Papua (10.96%), Gunung Rinjani in Nusa Tenggara (10.8%), and Manusela in Maluku (10.59%). Tanjung Puting, Rawa Aopa Watumohai, Meru Betiri, Manusela, and Lorentz also experienced the highest increase in their buffer areas, with 63.7%, 12.48%, 11.7%, 11.2%, and 10.2% respectively. Other buffer areas with high HFs are Laiwangi Wanggameti in Nusa Tenggara (16.3%) and Berbak in Sumatra (13.8%, Table 1).

**Figure 5:**
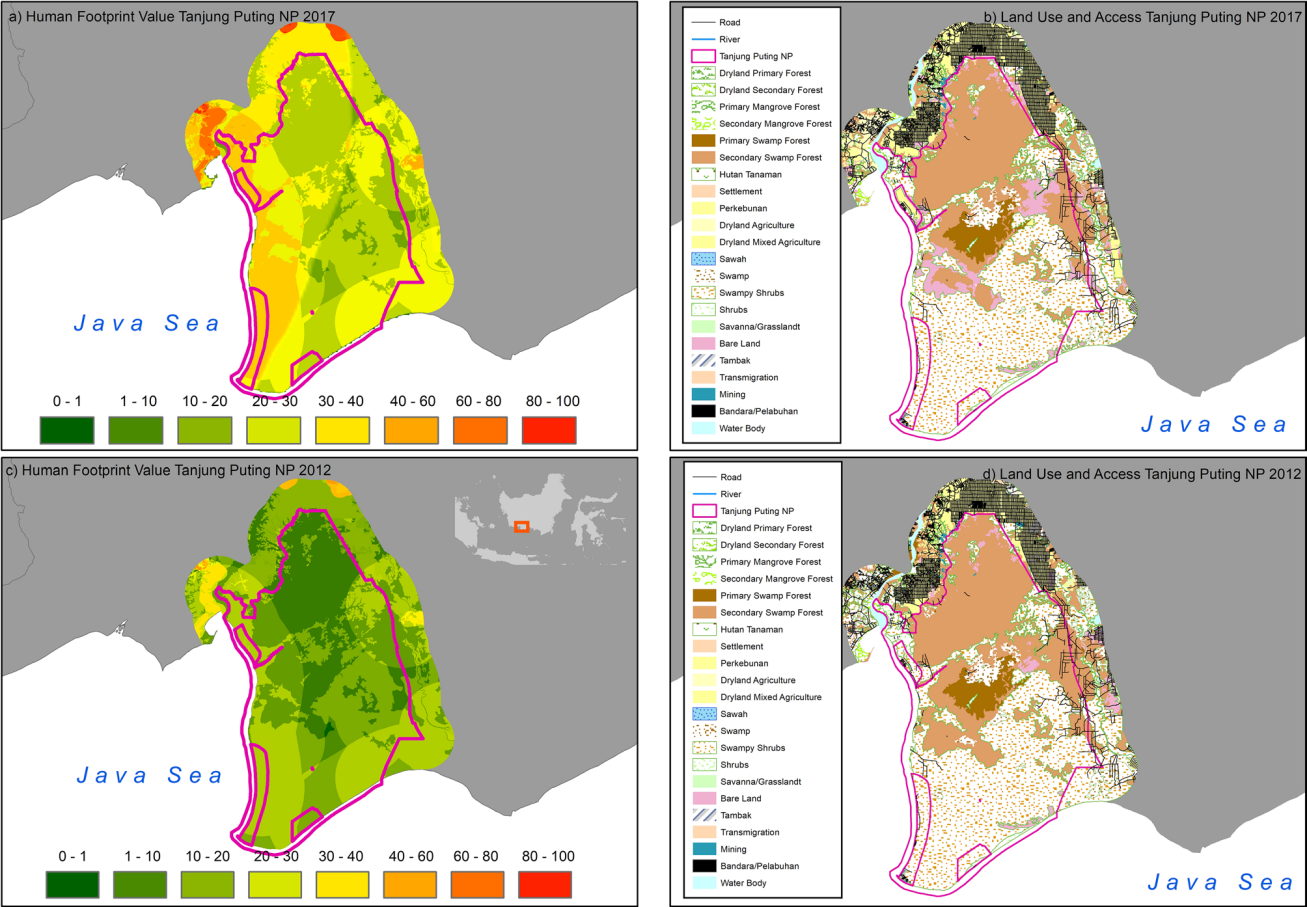
Human Footprint and land use in Tanjung Puting National Park, Kalimantan in 2012 and 2017. Activity data maps of Tanjung Puting NP (land cover maps, roads, river and settlements of respected years) used in the Human Footprint analysis in this study were obtained from the Indonesian Ministry of Environment and Forestry^32^ and Binamarga^33^ and NP boundary was obtained from The World Database on Protected Areas (https://www.protectedplanet.net/en). The Maps were created using Esri ArcMap 10.8 (https://www.arcgis.com).

Tanjung Puting NP in Kalimantan (Fig. 5) experienced the greatest HF increase, both inside and outside the park. Tanjung Puting also experienced land transformation such as secondary swamp forest (peatland) becoming degraded due to agriculture and plantation expansion, illegal logging and forest fires^35^.

### Jawa Bali bioregion

Jawa Bali is the most populous and well developed bioregion in Indonesia, hence it also has the highest HF compared to the other bioregions. However, in the study period between 2012 and 2017, total forest cover in NPs in Jawa Bali actually increased by 1.23%^21^. In those 5 years, all other bioregions experienced a decrease in forest cover in NPs, ranging from 0.24% in Maluku to 2.34% in Kalimantan^21^. There are 11 NPs in the Jawa Bali bioregion, four of which experienced a decrease in HF both inside and outside the parks. Jawa Bali bioregion also had higher increases in the HF outside the parks compared to the inside (Fig. 4).

Human population is the highest driver of HFs in Jawa Bali, both inside the parks and outside. Access and roads are the second biggest driver inside the parks, followed by IP, agriculture, and OLU. In the buffer areas, roads are the second highest, followed by access, agriculture, IP and OLU.

## Discussion

### Drivers of the human footprint in and around national parks

It has been recognized that to be able to achieve their management objectives, PAs must also be supported by appropriate management of the surrounding areas. Changes to the environment in areas directly adjacent to PAs are especially important in determining the ecological conditions for biodiversity and habitats within them^36^. In the past, conservation areas were managed as self-contained units, with institutional management, day to day management of species and habitats, and research all taking place within the boundaries of PAs regardless of activities outside. Protected areas were considered to be free from encroachment by economic and human development. However, PAs are embedded in landscapes and seascapes, which are complex social-ecological systems shaped by interactions between biological, ecological and physical features mediated by the actions of people^37^. There are clear interdependencies between a PA and the surrounding social-ecological system^38,39^. Forest cover, human population, road expansion, and traffic within PAs reflect what is happening in the surrounding area^36^. These conditions are shown clearly in most of Indonesia’s NPs, as statistical analysis here showed that increased HFs inside NPs follow what is happening in the 10 km buffer areas.

- **Roads and access**

Terrestrial NPs in Indonesia are strongly impacted by infrastructure development, especially roads. Roads are the principal driver of HFs outside the parks in most bioregions, thus causing access (from roads) to be the principal driver inside the parks. Roads are generally built to support increasing human populations and new or expanding settlements^9^, but road construction within or adjacent to PAs places high pressures on them because it increases the rate of forest fragmentation and the loss of habitat for wildlife^36^. Unfortunately, road access also helps people to encroach on NPs, although they also encroach on them using natural access via rivers and along coastlines, and facilitate agricultural and plantation development in and around NPs^9^. In addition, road constructions can cause erosion and landslides^36^.

Although they differ in the degree of pressure in each bioregion, roads and access are still the highest drivers of HFs. NPs in Jawa Bali, Nusa Tenggara, Maluku and Sulawesi have suffered the most from road development, both inside the parks and adjacent to them. Studies have shown that planned and ongoing road and rail-line developments currently occurring in Kalimantan will likely have many negative ecological impacts, including fragmenting large expanses of intact forest. If all planned projects proceed under the assumption that new road and rail projects will have only a 1-km buffer on either side, landscape connectivity across the region will decline sharply from 89 to 55%^40,41^. In Sumatra, a planned 2700-km pan-Sumatra Highway would affect forests in and around Kerinci Seblat and Leuser NPs. The highway would provide opportunities for encroachment into the edges of these parks^42^. A study in Rawa Aopa Watumohai NP in Sulawesi, documented one of the highest levels of road mortality in Asia. The survey was conducted along a 21.8-km section of paved highway that crosses the park. Forty taxa, 15% of which are endemic to the Wallacea region, were represented by animals killed on this stretch of road^43^.

It is believed that similar results will occur in other bioregions such as Nusa Tenggara, Maluku and Papua. In order to reduce the impact of road developments and access to NPs, it has been emphasized that prevention, or at least close supervision, of road construction within and adjacent to PAs is very important in increasing management effectiveness^44^. However, new Indonesian regulations on road developments seem to provide weaker protection for protected forests which are less known and less reported in the media, but contribute significantly to biodiversity conservation. These kinds of forests, less known but critical for conservation, usually are the very kinds threatened by infrastructure expansion, especially road developments^42^.

b. **Human population**

After roads and access, human population is the main driver of HFs both inside and in the buffer areas of Indonesia’s terrestrial NPs. Human population is frequently mentioned as the main cause of declines in the quality of nature, with higher human populations leading to more adverse impacts on nature. Human population increases in areas adjacent to PAs add more pressures both inside the PAs and within the buffer areas around them^24^. The changes in human pressures in PAs and surrounding areas in Sundaland seemed to correspond with the increases in human population^45^.

This study also showed that it was the case for 43 terrestrial national parks in Indonesia. Tanjung Puting NP in Kalimantan has the highest increase in the HF and it was mostly due to a large local increase in human population. It was 21.31 people/km^2^ in 2010, rising to 24.7/km^2^ in 2015, an increase of 15.96%^1^. The higher the human population, the more natural resources are needed to support it. However, the impacts of the interaction between human populations and natural environment are not yet known in detail, partly because the impact will depend on the nature of the interaction as well as on the type of ecosystems, or specific natural processes^22^. This is also the case in Tanjung Puting NP where the very high increase in the HF in the park is due to changes in the indirect effect of increasing the human population outside the parks.

c. **Land transformation**

In this HF study, land transformation comprises agriculture, industrial plantations (IP), built up environment (BUE) and other land uses (OLU) such as bare lands and shrubs. Terrestrial NPs in Indonesia experienced these land transformations both inside and outside the park areas. While land transformation inside the parks will certainly have direct impacts on forests and biodiversity, land transformations occur adjacent to park areas can also have detrimental effects. In Indonesia, land transformation around PAs due to human population changes, or regional expansion followed by infrastructure needs, mobility, mining, broad-scale plantations, as well as market demand for certain commodities, have been identified as the pressures that affect the condition of PAs^2^. PAs usually mirror environmental degradation that occurs in the surrounding areas. A decrease of natural habitats in the buffer areas has led to a decrease in the ability of PAs to protect their biodiversity. This has happened in Way Kambas NP, Sumatra. Between the early 1980s and 2011, the percentage of forest cover to a distance of 50 km around Way Kambas National Park in Lampung, Sumatra, fell from 80 to 33%^46^, while the park itself lost around 8% of its forest cover only within 5 years from 2012 to 2017^21^.

Using production forest or industrial plantation (IP) concessions as buffers for conservation areas can increase the effectiveness of management to protect forests and biodiversity^47^. However, this is not always the case for Indonesia’s national parks. For example, Tesso Nilo in Sumatra has industrial timber production forest and palm oil plantations as buffers^48^, however its HF increased more inside the park (19.1%) than in the buffer zone (7.5%) between 2012 and 2017 (Table 1). Other land use such as bare lands and shrubs are still a big part of HF drivers in Indonesia’s NPs. There are around 2.2Mha degraded forest inside PAs in Indonesia. This is about 10% of the total areas of PAs^7^. Tanjung Puting NP in Kalimantan suffered the highest increase of HF between 2012 and 2017. This was partly due to land transformation in the form of natural peatlands degradation. Most peatlands in Indonesia, including in Kalimantan, have experienced decades of mis-management. Peat swamp forests have been subjected to illegal logging as well as plantation and agricultural expansion. This has led to peat being drained with a loss of biodiversity and a significant increase in fire vulnerability. In 2015, coupled with a strong El Niño event, extensive fires burned thousands of square km of peatlands, including in Tanjung Puting^35^.

### Changes in the human footprint between 2012 and 2017

Most of the terrestrial NPs in Indonesia experienced increase of HF both inside and outside the parks between 2012 and 2017. Among 43 NPs, only 4 NPs in Jawa Bali bioregion have lower HF inside and outside the parks. Danau Sentarum in Kalimantan and Komodo in Nusa Tenggara have lower HF only inside the parks. In 2015, there were changes in the Indonesia’s Ministry of Environment and Forestry that caused the reduction of the number of forest guards on each NP in 2012 compare to 2017. Law enforcement policy plays an important role in reducing forest degradation and deforestation^49,50^. Although directly reduced the number of forest guards, the overall organizational changes were meant to achieve better protection and conservation outcomes not just in protected areas. However, in terms of forest cover protection, these changes have not yet seen positive impacts. Between 2012 and 2017, forest cover in most of Indonesia’s terrestrial NPs was still declining, although not significantly related to NPs forest guards’ reduction. Reduced forest cover was significantly caused by increase of HF inside the parks^21^.

The effectiveness of conservation areas in stopping forest encroachment, or at least reducing deforestation, hunting, and forest fires, is directly related to management activities. Basic management activities such as enforcing regulations, defining clear boundaries, and providing direct compensation to local communities around parks, have succeeded in increasing management effectiveness^51^. Increased investment in management infrastructure, including increased numbers of personnel, is important to maximize forests and biodiversity protection^52^. Partnership and collaboration with many parties can also create success stories. One example is the Bunaken Marine National Park in North Sulawesi, which collaborates with local communities, the diving tourism industry, and provincial and district level governments. Through this partnership the sustainability of revenues for national parks, community involvement and support and the achievement of conservation efforts has been increased^53^. Kayan Mentarang NP in Kalimantan also benefits from local communities involvement in PA management and has the lowest HF in Indonesia. This 1.4 Mha of forest is the largest forest conservation area in Kalimantan. The Human Footprint in Kayan Mentarang is low because of the difficult access due to steep terrain and wild rapid rivers^54^. Such areas are less suitable for agriculture or plantation expansions^29^. Besides that, the traditional Dayak communities living in Kayan Mentarang still value natural ecosystem highly and that helps to protect them from external influences^55,56^. Management by indigenous peoples or local communities can be a factor that improves the effectiveness of conservation area^57^. Other success stories involving communities’ collaborations also happened in Jawa Bali bioregion.

### Jawa Bali bioregion

Jawa Bali bioregion is special because among the 43 terrestrial NPs in Indonesia, 6 in Jawa Bali managed to increase their forest cover despite the highest HF within and outside the parks. In a modeling study, it was suggested that in 100 years, 6 to 8 billion people on Earth will mostly be living in urban areas, with very low number still living in extreme poverty, and almost all participating in a technologically driven, interconnected market economy. They explained that the interacting effects of urban lifestyles on fertility, poverty alleviation, and ideation will shift the dynamics of the main drivers of deterioration in nature. At that stage, conservation practice will transform itself from a discipline managing declines in nature to a transformative movement of recovery^58^. This model is in line with another study that showed Indonesia’s deforestation rates will be declining after 2030 and will reach nil deforestation and change to afforestation in 2035–2045^59^. This may have already started to happen in the Jawa Bali bioregion. NPs in all Indonesia’s bioregions experienced reduced forest cover from 2012 to 2017, except in the Jawa Bali bioregion^21^. National parks in Jawa Bali had the highest HF outside and inside the parks. However, forest cover was maintained, with some NPs even managing to gain forest cover, over the 5 year period^21^. The ability to regenerate forests will increase if the pressure of human activity decreases^60^. Most NPs in the Jawa Bali bioregion serve as the main water sources for the surrounding areas, hence are crucial to people's lives. So although the HF in the buffer zone may be high due to the expansion of agricultural land, settlements, road construction, and other human activities, the parks’ forests are still maintained for the sake of water availability^61^. Furthermore, the remaining forests of Jawa Bali NPs occur in the steep mountains, making it difficult to encroach. The more accessible landscapes are already degraded^62^.

Social and cultural customs in Jawa Bali might also be contributing to this promising phenomenon. The Balinese have a number of local policy values that govern human relations with nature. Some examples that continue to be carried out today are the tree sacred belief system and the Hindu philosophy of Tri Hita Karana. Customary laws to protect trees are not just related to certain sacred species like banyan trees^63^ but also to any trees in sacred areas such as waterfalls, used by Balinese Hindus to perform prayer activities, meditation and activities related to the spiritual^64^. Tri Hita Karana teaches that harmony between humans and nature is one of three sources of human well-being^65^. In Java, there is the Pranata Mangsa system which is a calendar of traditional seasonal rules that govern natural resource use. This is useful as a cultural aid in the preservation of the natural environment^66^. One of the uses of Pranata Mangsa today is the Brubuh practices related to forest preservation. Brubuh is a traditional seasonal logging practice. It is conducted by only cutting down trees in certain seasons according to Pranata Mangsa. Using this practice, the forest can be better protected^67^. Support of this kind from various sources, like in Jawa Bali bioregion, Kayan Mentarang and Bunaken, improves the effectiveness of the management of PAs^57,68^.

## Conclusion

Human footprint analyses in this study showed that PAs, especially terrestrial national parks, in Indonesia, have experienced impacts from increased roads and access, human population as well as land transformations. Protected areas usually mirror environmental degradation that occurs in the surrounding areas. Therefore, it is very important to maintain the ecological environment around a conservation area^36^. A balance between conservation and sustainable development might be obtained by integrating the conservation area with the surrounding area^44^. Collaboration from local communities and indigenous people, especially in utilizing environment services, also proved to be beneficial to protected areas. More support for these kinds of management activities throughout Indonesia would help mitigate the increasing HFs detected in this study and perhaps begin to shift momentum towards the kind of recovery now evident in the Jawa Bali bioregion.

## Methods

**Figure 6:**
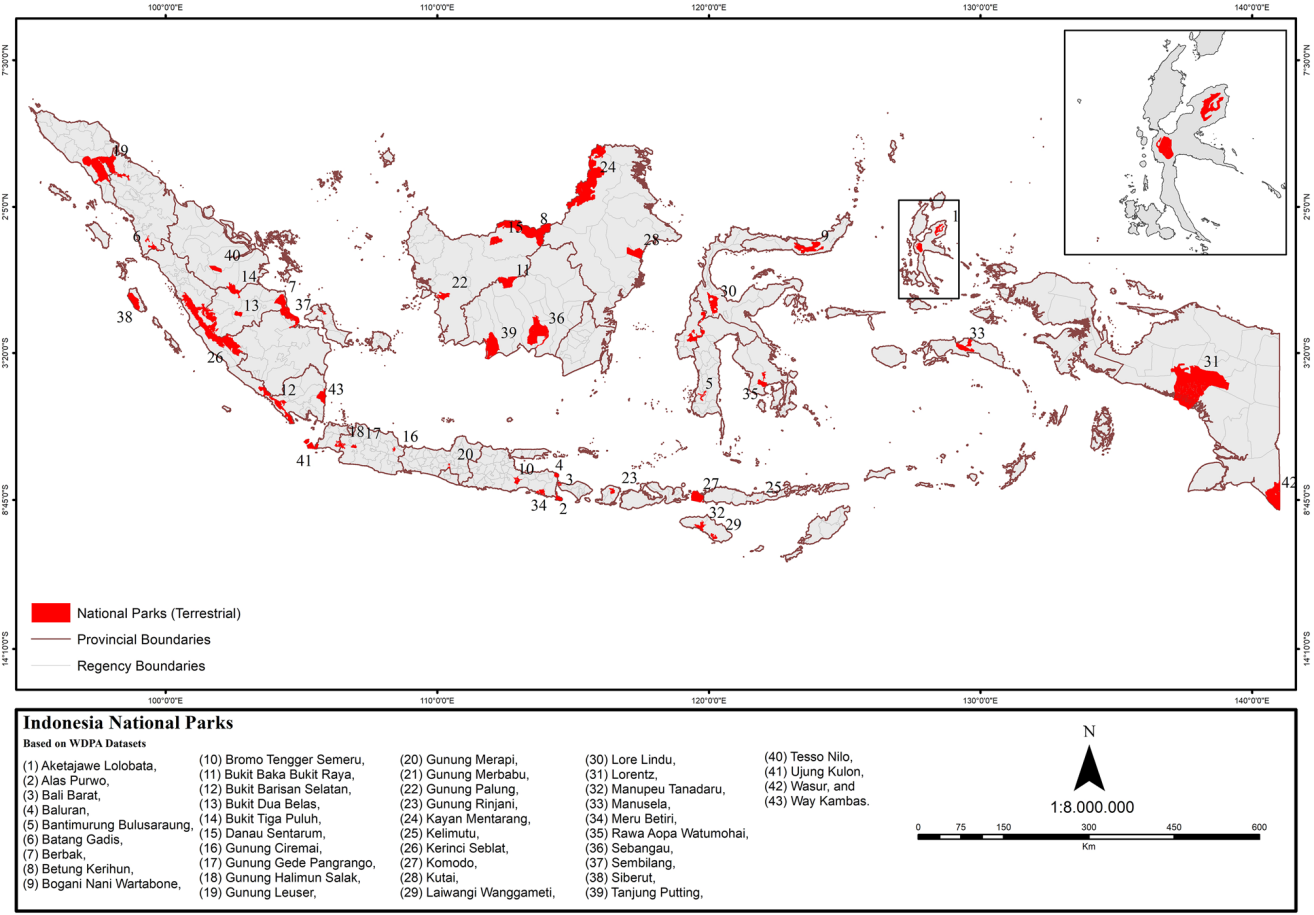
Forty three (43) terrestrial national parks in Indonesia included in the analysis. National parks boundaries were obtained from The World Database on Protected Areas (https://www.protectedplanet.net/en). The Map was created using Esri ArcMap 10.8 (https://www.arcgis.com).

The Human Influence Index (HII) was measured to quantify the pressures on national parks both within the parks, and within a 10 km buffer outside the parks. The HII scores were then converted into a Human Footprint (HF) map^22^. We used the 43 terrestrial national parks established up to and including 2012 (Fig. 6). They are distributed across 7 bioregions: Sumatra, Kalimantan, Sulawesi, Nusa Tenggara, Maluku, Papua and Jawa Bali^69^.

**Figure 7:**
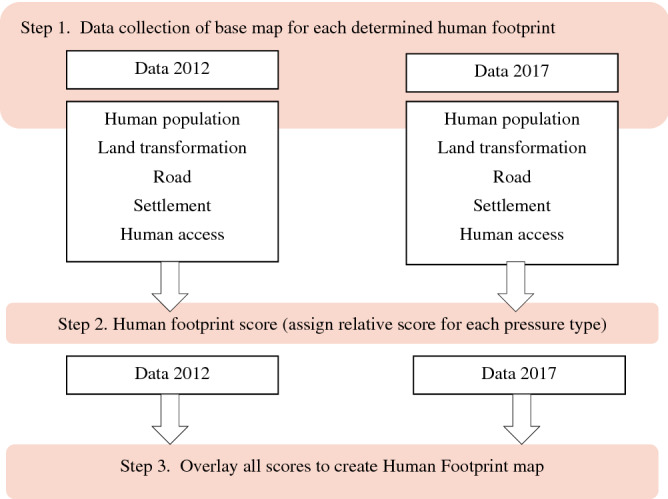
Flowchart showing the process of mapping the Human Footprint in 43 terrestrial national parks in Indonesia and in a 10-km buffer surrounding them. The steps detailed were slightly modified from the original^70^.

The steps to obtaining HII scores are shown in the diagram in Fig. 7, following a study on global HFs^70^. First, the different types of human activity that cause changes in forest cover were determined. Modification of the classification of pressure types from the original ones^22^ is shown in Table 2. Sub-pressures shown in Table 2 and scores were modified based on conditions in Indonesia and data availability as follows. In Indonesia, man-made environments such as agriculture and plantation areas usually have higher human activities due to the use of more human power compared to global averages. Thus HII scores in those areas were increased from the original scores^22^. In this study, roads and settlements were divided into more detailed types and given scores more closely related to each type (Table 2). Besides that, in some NPs, certain habitats are natural while in others the same habitat types occur due to human disturbances. Scrub and shrub habitats usually result from forest lost and deforestation, except in NPs with savanna-like natural habitats such as in East Jawa and north-west Bali. In order to correctly score natural habitats versus non-natural habitats, habitat conditions were assessed relative to a 1990 National Parks habitat map. The NPs studied here were established and had legal status between 13 and 37 years ago. Habitats shown on the 1990 map were deemed to be natural and therefore not much disturbed by human activities. Hence, any areas in which habitats have stayed the same since 1990 were considered to be natural and given a score of 0.

**Table 2:** Relative scores for pressures inside national parks and in 10 km buffers outside the parks used in the Human Footprint analysis.

Pressures	Sub-pressures	Scores		Data sources
		This study	Sanderson et al., 2002	
Human population	–	0–10	0–10	UN WPP-*Adjusted Population Density* 2010 and 2015^23^
Land transformation	Built-up environment (mining, airport, transmigration area)	10	10	Landuse thematic map 2012 and 2017^32^
	Agriculture (plantation, dryland agriculture, mix agriculture, paddy field)	9	6–8	
	Plantation (industrial plantation, palm oil plantation)	9	6–8	
	Other land use (bare land, shrub, swamp shrub, fishpond)	6	4	
	Natural habitat (primary forest, secondary forest, savanna, other natural biomes)	0	0	
Road The score is given to the road networks and all areas up to 2 km from roads	Toll road	8	8	Binamarga Map 2014^33^
	National road	8		
	Province road	6		
	District road	4		
	Other road	2		
Settlement	Province capital city	10	8–10	Landuse thematic map 2012 and 2017^32^
	District capital city	8		
	Sub-district capital city	6		
	Other settlement	4		
Human access The score is given to all areas up to 15 km from railways, big rivers and coastlines	Railway	4	4	Binamarga map 2014^33^; Landuse thematic map 2012 and 2017^32^
	Big river	4	4	
	Coastline	4	4	

Base maps used for each pressure data were in a 30 m × 30 m pixel format except for the human population density map^25^. The human population density map has a 1 km × 1 km pixel format. For this analysis, the population density map data was reduced to 30 m × 30 m using the ArcGIS bilinear sampling method^70^. In order to create the global Human Footprint maps, the original human population data^23^ were re-scaled and standardized to a maximum value of 10^22^. After all sub-pressures were scored, each score was summed for all pixels in each base map. All data source base maps were then overlaid and all pixel values in the overlay maps were added up. This generated a total pressure value or total HII for each national park. Total HII scores were calculated for all parks and the 10-km buffer areas outside the parks. Human Influence Index (HII) is then described as a Human Footprint (HF) map. In order to create the HF map, the total value of each overlaid pixel was then standardized to a range from 0 to 100 using the formula:

$$HF_{i} = \frac{{\left( {HII_{i} {-} \, HII_{minj} } \right) \, \times \, 100}}{{HII_{maxj} - \, HII_{minj} }}$$

where i represents the pixel and j represents the national park of which the pixel is a member^24^.

The global scale Human Footprint^22^ was revised^70^. That revision used a total of 3114 sample points across the globe to validate the global human footprint map. For this study, 100 sample points were taken from 43 national parks. When comparing the world land area of 51,000,000 km^2^ with the land area of Indonesia covering 1,905,000 km^2^, then 100 sample points from 43 national parks was more than sufficient for validation purposes.

The validation was conducted by repeating the relative scoring for each pressure seen at the 100 sample points. The scoring was done visually by looking at land cover across an area of 1 km × 1 km at each sample point on high-resolution satellite imagery from Google Earth. Each area of 1 km × 1 km was allocated a dominant land cover (> 50%) and given a pressure score of 0–10, according to the researchers' visual interpretation and based on the pressure scores listed in Table 2. Validation of scoring was calculated using the non-parametric statistical method Root Mean Square Error^71^ and Cohen kappa statistics of agreement^72^ commonly used in geospatial analysis, especially when calculating the validity of land cover classifications. The relative score used in the model are valid if the Kappa Statistics are > 0.5. Validation analyses were conducted for both 2012 and 2017 human footprint data. Human Footprint or HII scores were then divided by the total area of each NP and its buffer area to get HII per ha. Percent changes of this HII per ha were calculated by comparing HII in 2012 and in 2017. Finally, The relationship between the percent change of HF between 2012 and 2017 in the 10-km buffer areas outside the parks and inside the parks was tested by simple linear regression analysis performed using the R statistic packages^73^.

## Data Availability

Data available upon request.
